# Case Report: A Novel Homozygous Frameshift Mutation of the *SKIV2L* Gene in a Trichohepatoenteric Syndrome Patient Presenting With Short Stature, Premature Ovarian Failure, and Osteoporosis

**DOI:** 10.3389/fgene.2022.879899

**Published:** 2022-04-27

**Authors:** Minyi Yang, Yu Jiang, Xinyu Shao

**Affiliations:** ^1^ Department of Endocrinology and Metabolism, The First Affiliated Hospital of Soochow University, Suzhou, China; ^2^ Department of Endocrinology and Metabolism, Dushu Lake Hospital Affiliated to Soochow University, Suzhou, China

**Keywords:** trichohepatoenteric syndrome, intractable diarrhea, premature ovarian failure, *SKIV2L* gene mutation, case report

## Abstract

**Background:** Trichohepatoenteric syndrome (THES) is a rare Mendelian autosomal recessive genetic disease characterized by intractable diarrhea, woolly hair, facial abnormality, immune dysfunction, and intrauterine growth restriction. THES mutations are found in the *TTC37* and *SKIV2L* genes, which encode two components of the human superkiller (SKI) complex.

**Methods and results:** We report one case of a 32-year-old woman of Chinese descent with THES, who was born with a low weight (2000 g). She had intractable diarrhea during the neonatal period and was allergic to cow’s milk and condensed milk, but did not require total parenteral nutrition. She experienced menarche at age 12 and amenorrhea at age 28. In May 2019, the patient presented with a left fibular head fracture and was diagnosed with osteoporosis. Genetic testing showed a novel mutation in exon1 [p.E5Afs∗37 (c.12_13del)] of *SKIV2L*, which is composed of 28 exons. After the diagnosis, hormone replacement therapy was prescribed, in addition to the routine calcium and vitamin D supplements.

**Conclusion:** This case expands the clinical characteristic and phenotype spectrum of THES, providing further understanding of *SKIV2L* and its autoimmune influence.

## Introduction

Trichohepatoenteric syndrome (THES) or syndromic diarrhea (SD) is a very rare and severe Mendelian disease [OMIM: 222470 (THES1, syndromic diarrhea) and 614602 (THES2)]. To date, only eight patients of Chinese descent have been reported to have THES in both English and Chinese literature (Chong et al., 2015; Lee W.-I. et al., 2016; Lee W. S. et al., 2016; Zheng et al., 2016; Chen and Shi, 2017; Zhang et al., 2021). The global estimated prevalence of the disease is 1/1,000,000 (Fabre and Badens, 2014). THES was first described by Stankler et al. (1982) and later identified by Girault et al. (1994) as a clinical entity. THES typically presents with severe, intractable diarrhea in the neonatal period, which leads to failure to thrive and short stature, woolly and brittle hair, facial abnormality characterized by broad nasal root and prominent forehead and cheeks, immune dysfunction, and intrauterine growth restriction. Furthermore, liver disease, mild intellectual disability, café au lait or dyschromic spots, and congenital heart disease may be detected to a lesser extent in patients with THES (Fabre et al., 2018).

THES is often associated with tetratricopeptide repeat domain 37 gene (*TTC37*), which was first confirmed to be a causative gene in 2010 (Hartley et al., 2010), and superkiller viralicidic activity 2-like gene (*SKIV2L*), which was subsequently considered different and independent from *TTC37* (Fabre et al., 2012). *TTC37* was mutated in about 68% patients whereas *SKIV2L* was mutated in the remaining 32% (Bourgeois et al., 2018). The two genes encode two proteins that are components of the human superkiller (SKI) complex, which is a heterotetrameric cytoplasmic cofactor of the RNA exosome. The human SKI complex contains hSKI2 (*SKIV2L*), hSKI3 (*TTC37*), and hSKI8 (*WDR61*) (Kogel et al., 2022). Mutations in *TTC37* and/or *SKIV2L* may lead to dysfunction of the SKI complex, thereby, disrupting mRNA regulation and nonfunctional mRNA decay (Fabre et al., 2012). However, the specific mechanism of the disease remains unexplored.

Here, we report a special case of THES with premature ovarian failure, growth failure, and osteoporosis, who had syndromic diarrhea during the neonatal period.

## Materials and Methods

### Clinical Examinations

The medical history of the proband, including clinical manifestations and therapeutic actions, was provided by the patient and her mother. Physical examination, laboratory tests, and imaging test scans, including ultrasound and pituitary magnetic resonance imaging, were performed.

### Peripheral Blood Collection and High-Throughput Sequencing

Genomic DNA was isolated from the peripheral blood obtained from the proband, her mother, and her brother and was fragmented to an average size of 150 bp using an S220 Focused-ultrasonicator (Covaris, Massachusetts, United States). High-throughput sequencing was performed by MyGenostics (Beijing, China) using the Illumina HiSeq X ten system. After sequencing, the raw data were saved in the FASTQ format. Both Illumina sequencing adapters and low-quality reads (<80 bp) were filtered using the cutadaptor software (Kechin et al., 2017). The clean reads were mapped to the UCSC hg19 human reference genome using the BWA software (Li and Durbin, 2009). The duplicated reads were removed using Picard tools, and the mapped reads were used for the detection of variations. The variants of single nucleotide polymorphism (SNP) and small insertions/deletions (INDEL) were detected using HaplotypeCaller in the GATK software (Van der Auwera et al., 2013) and filtered using VariantFiltration in the GATK software, according to the following criterions: 1) variants with mapping qualities <30; 2) the total mapping quality zero reads <4; 3) approximate read depth <5; 4) QUAL <50.0; 5) phred-scaled *p*-value using Fisher’s exact test to detect strand bias >10.0. The data were then transformed to the VCF format. Variants were further annotated using the ANNOVAR software (Wang et al., 2010) and associated with multiple databases, such as 1000 genome, ESP6500SI, dbSNP, EXAC_ALL, Inhouse (MyGenostics), HGMD, and ClinVar. The interpretation of sequence variants was performed according to the guidelines from the American College of Medical Genetics and Genomics (ACMG) (Richards et al., 2015). Furthermore, polymerase chain reaction (PCR) amplification and Sanger sequencing were carried out on DNA samples from the proband as well as from her family members to identify co-segregation of the disease phenotype and the detected variants, as described previously (An et al., 2015). The following primers designed for *SKIV2L* were used: Forward: 5′-AAG​TTG​CCT​CTA​CTT​CCG​CC-3′, Reverse: 5′-AAT​GCG​GGT​CAA​AGG​TTA​GG-3′. The PCR products were sequenced using an ABI3730XL DNA analyzer (Applied Biosystems™; Thermo Fisher Scientific Inc., Waltham, Massachusetts, United States), according to the manufacturer’s protocols. The results were compared with the NCBI reference sequence, NM_006929.5, to identify nucleotide substitutions.

## Results

### Case Presentation

Herein, we report about a woman of Chinese ethnicity who presented with short stature, amenorrhea, and osteoporosis at the age of 30 years.

**FIGURE 1:**
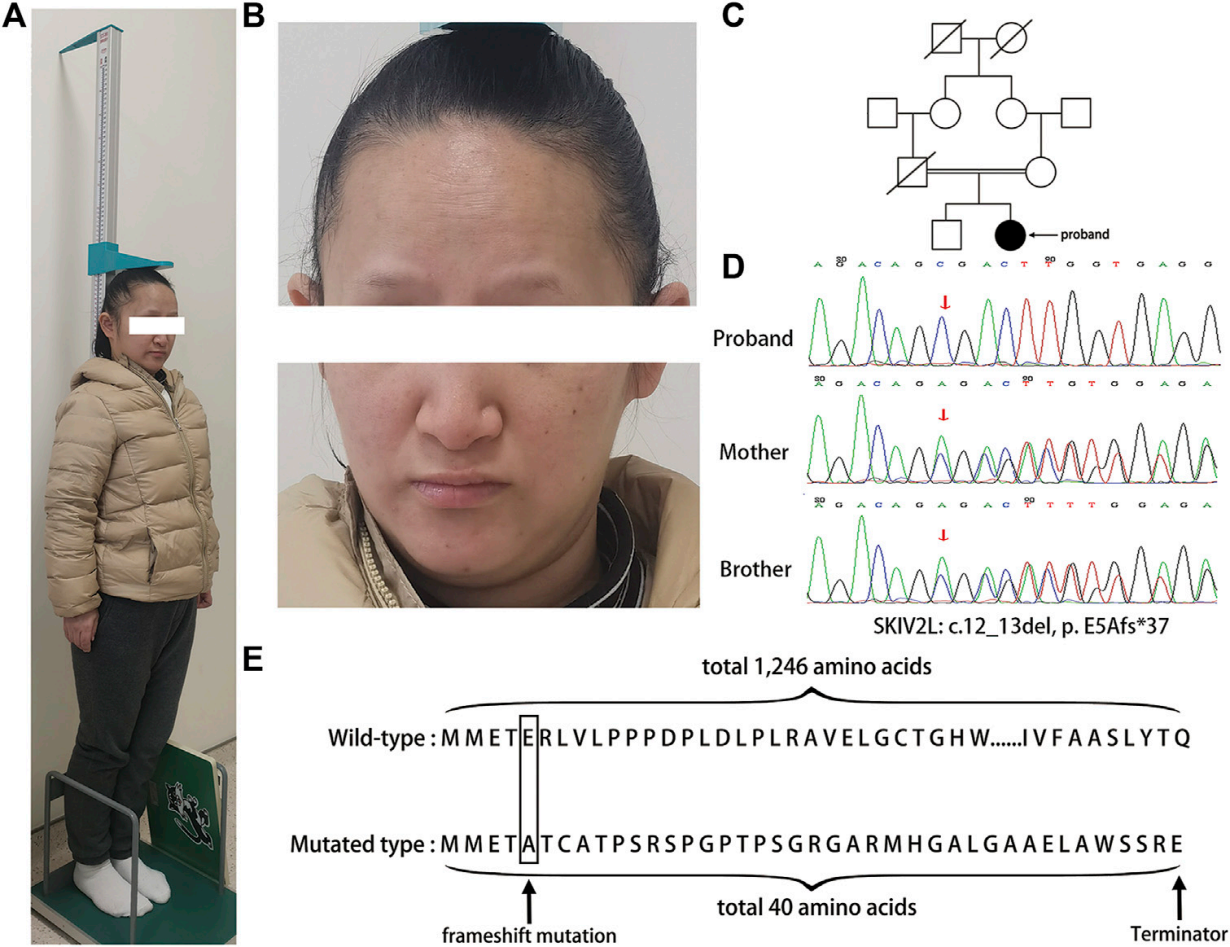
**(A)** The image shows that the proband was short in stature. **(B)** The graph shows that the patient had facial dysmorphism. **(C)** Pedigrees of the family diagnosed with trichohepatoenteric syndrome. **(D)** Sanger sequencing chromatograms of DNA samples from the proband and her mother and younger brother showing the autosomal recessive mutation, c 12_13del in *SKIV2L*. Arrows indicate the position of the mutation. **(E)** Amino acid sequences of wild-type and mutated *SKIV2L*. The mutation of *SKIV2L* results in a truncated protein composed of 40 amino acids, compared with the wild-type *SKIV2L*, which is composed of 1246 amino acids.

She was born at term, weighing 2000 g (<3rd percentile) at birth, making her a small for gestational age (SGA) infant. She was the first child of consanguineous parents (Figure 1C), and has no significant family history. She suffered from chronic intractable diarrhea (4–5 times per day, about 10–50 g each time) during her neonatal period without vomiting after ingestion. The Bristol stool form value was 5. The amount of breast milk was low, but the patient had an episode of anaphylaxis to cow’s milk protein formula and condensed milk. Although chronic diarrhea persisted, the symptom gradually improved in the past 10 years. Her growth rate was <5 cm per year. She was treated with growth hormone injections for short stature for 1 year. The patient had a body height of 1.35 m [<3 standard deviations (SDS)] and body weight of 34 kg (<2 SDS) on admission. The height of her father and mother was 1.70 and 1.63 m, respectively. The body mass index was 18.65 kg/m^2^ (normal range 18–24 kg/m^2^), which is close to the lower limit of the normal value. According to the Corrected Midparental Height the normal height of the proband should be 1.60+/−0.05 m. The patient presented with mild intellectual retardation that did not affect oral communication. She visited the primary school and has been working in a factory. Brother of the proband, whose height is 1.80 m, is normal both physically and intellectually.

The patient experienced menarche at age 12 with a normal menstrual cycle of 28 days, and her menstrual period lasted 5 days. However, after a few years of regular menstruation, her cycle became longer, and she finally progressed to menopause at the age of 28 years. In May 2019, the patient had an accidental fall and got a left fibular head fracture. After surgery to correct the fracture, she appeared to have common peroneal nerve damage, resulting in drop foot.

We observed certain malformations, such as woolly hair, broad nasal root and prominent forehead, in the patient, and she had mild exophthalmos (Figures 1A,B). There was no history of previous abdominal surgery, radiotherapy treatment, or exposure to toxic metals. The results of routine blood tests, liver and kidney function, thyroid function, blood sugar, glycosylated hemoglobin (5.3%), insulin-like growth factor-1 (127 ng/ml), and electrolyte tests were normal. Karyotyping was 46, XX. Hormonal profiles are shown in Table 1. Ultrasounds of the heart, abdomen, and urinary system were normal. Ultrasound examination indicated that the size of her uterus was 32 × 27 × 33 mm (normal size, 70–80 × 40–50 × 20–30 mm), and endometrial thickness was 4 mm (normal range, 0.5–10 mm). The patient’s ovaries measured 14 × 12 × 11 mm and 14 × 10 × 10 mm (normal size, 40 × 30 × 10 mm), respectively. X-ray images showed epiphyseal fusion. DEXA bone mass density (BMD) showed low lumbar spine and femur bone density at 0.718 g/cm^2^ (normal range, 0.715–0.975 g/cm^2^) and 0.876 g/cm^2^ (normal range, 0.994–1.234 g/cm^2^), respectively. Magnetic resonance imaging indicated a pituitary microadenoma measuring 4 mm.

**TABLE 1:** Laboratory values of various sex hormones and liver function in the patient.

	Reference range	First test	Second test (17 months later)	Third test (21 months later)
LH (mIU/ml)	2.12–10.89	58.94	27.95	41.84
FSH (mIU/ml)	3.85–8.78	193.18	72.78	105.28
E2 (pg/ml)	27–122	<20	21.4	24.9
Prog (ng/ml)	0.31–1.52	0.54	0.27	0.34
PRL (ng/ml)	3.34–26.72	11.05	4.96	10.81
Testo (ng/ml)	0–0.75	0.33	0.33	0.43
AMH (ng/ml)	0.711–7.59	NA	NA	<0.01
AST (U/l)	13–35	14.7	18.6	NA
ALT (U/l)	7–40	13.8	20.7	NA
GGT (U/l)	7–45	18.3	21.1	NA
T-BIL (μmol/L)	3.4–17.1	11.3	9.2	NA
D-BIL (μmol/L)	0–6.8	3.7	2.3	NA
I-BIL (μmol/L)	1.7–10.2	7.6	6.9	NA

E2, estradiol; FSH, follicle-stimulating hormone; LH, luteinizing hormone; PRL, prolactin; Prog, progesterone; Testo, testosterone; AMH, anti-Müllerian hormone; AST, glutamic pyruvic transaminase; ALT, glutamic oxaloacetic transaminase; GGT, γ-glutamyl transpeptidase; T-BIL, total bilirubin; D-BIL, direct bilirubin; I-BIL, indirect bilirubin.

### Genetic Analysis

Whole-exome sequencing revealed a novel variant in exon 1 of *SKIV2L* [p. E5Afs*37(c.12_13del)], leading to a homozygous translation frameshift (PVS1) (Figure 1D). The variant was absent from the controls (ClinVar, HGMD, 1000g2015aug_all, ExAC_All, ESP6500SI) (PM2) and databases and was assessed as “likely pathogenic” using ACMG criteria (PVS1 + PM2). The proband’s father died due to cholangiocarcinoma and was unavailable for genetic testing, but her mother and brother underwent Sanger sequencing, which showed that they were heterozygous for the variant. No variant was detected in *TTC37*.

**FIGURE 2:**
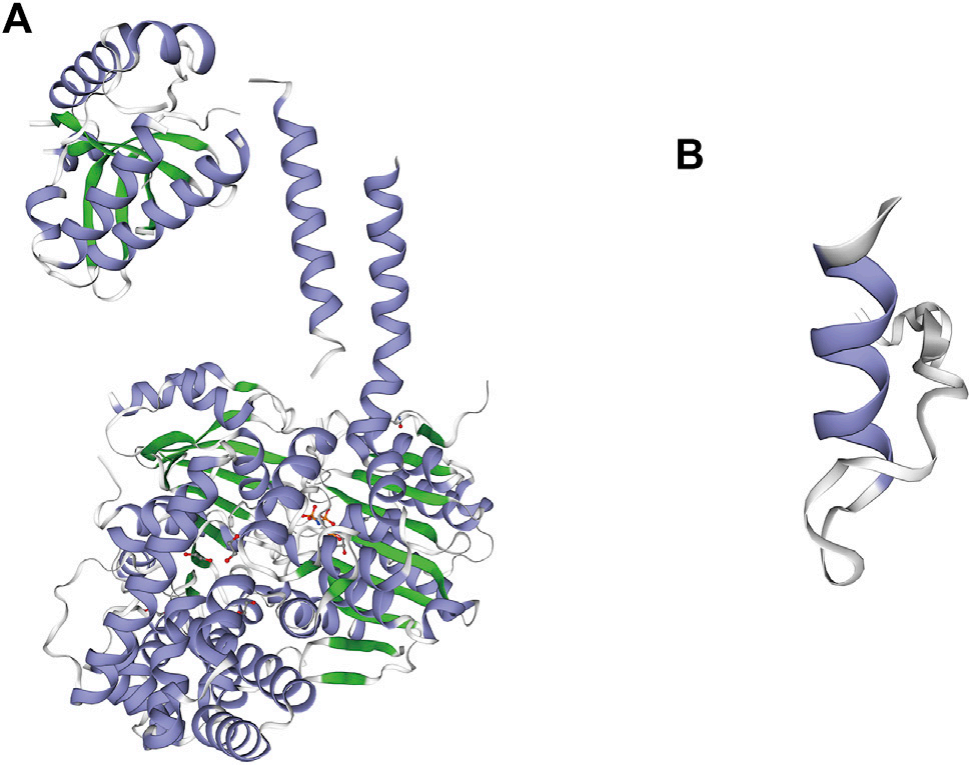
Difference between the wild-type and p.E5Afs*37 (c.12_13del) mutant protein of *SKIV2L*. **(A)** Three-dimensional model of wild-type *SKIV2L*. **(B)** Model of the mutant protein.

Three-dimensional models of the wild-type and p.E5Afs*37(c.12_13del) mutant protein of *SKIV2L* were generated by the SWISS-MODEL online server (Guex et al., 2009; Bertoni et al., 2017; Bienert et al., 2017; Waterhouse et al., 2018; Studer et al., 2020). Global model quality estimation (GMQE) and qualitative model energy analysis (QMEAN) for the mutant model of *SKIV2L* were 0.33 and −1.84, respectively, indicating the good quality of the model (Figure 2). The model shows that the mutation leads to complete damage of the RNA helicase subunit encoded by *SKIV2L* in humans, causing a truncated 40 amino acid protein (Figure 1E).

### Treatment and Prognosis

After THES diagnosis, hormone replacement therapy was prescribed, in addition to the routine intake of calcium and vitamin D supplements. During treatment with hormone replacement therapy, the patient had normal menstrual patterns. After treatment for over a year, we performed follow-up assessments and sex hormone re-examination (Table 1). Ultrasound results of the patient’s uterus and ovaries were nearly similar.

## Discussion and Conclusion

THES is a rare and severe Mendelian autosomal recessive disease, which is characterized by severe, intractable diarrhea (76/77), woolly and brittle hair (71/73), facial dysmorphism (66/67), severe immunodeficiency (48/67), growth failure, and mild intellectual disability. Liver disease (41/61), congenital heart disease (15/43), and abnormal platelet function are rarely observed. However, liver disease is diagnosed in about half of the patients (Fabre et al., 2018). The specific variants are observed in *TTC37* and *SKIV2L*. The *SKIV2L* mutation is less commonly found in patients, and its incidence was about 32% (Bourgeois et al., 2018). The two genes encode the human SKI complex subunits associated with RNA degradation. *SKIV2L* is a housekeeping gene composed of 28 exons, and its product (SKI2W) plays an important role in regulating the innate immune response to foreign nucleic acids (Eckard et al., 2014). However, there appears to be no “mutational hot spot” in either *TTC37* or *SKIV2L*. According to Bourgeois et al. (2018), mutations are found in almost every exon of the two genes. Whole exome sequencing analysis showed a new variant in exon 1 of *SKIV2L* [p.E5Afs*37 (c.12_13del)], leading to a translation frameshift and truncated protein, which was greatly shortened from 1,246 to 40 amino acids, predictably causing a loss of function. The global prevalence of the disease is extremely low, and only eight Chinese THES patients have been reported in the available literature to date.

Almost all patients diagnosed with THES showed intractable diarrhea during infancy. An absence of intractable diarrhea has been observed only in three cases (Rider et al., 2015; Karaca Edeer et al., 2019; Poulton et al., 2019). Diarrhea occurs in almost all cases, varying from mild to severe. Our proband presented with chronic intractable diarrhea and an allergy to cow’s milk and condensed milk. Since the symptoms of diarrhea were mild, which is not often seen in THES patients, the patient did not accept total parenteral nutrition or a hydrolyzed protein formula (Fabre et al., 2018). Because the symptoms gradually improved, the patient did not undergo an intestinal biopsy; hence, we do not know whether the diarrhea was caused by chronic inflammation in the gastrointestinal tract, which may result from an autoimmune influence. We consider poor absorption and malnutrition to be the main reasons for her short stature (1.35 m). The proband is one of the few patients who has lived to 30 years of age. Considering that the diarrhea was mild, no serious organ involvement and immunodeficiency was presumed. It is noted that the specific symptoms of our proband differed from those of previously reported cases (Table 2). As most cases are from Europe, these differences may be attributed to natural physiological differences between races.

**TABLE 2:** Comparison of clinical features of the proband with a large cohort of patients with THES.

	Entire THES cohort (*n* = 80) (Fabre et al., 2017)	THES with *SKIV2L* mutation (*n* = 14)	Patient
Sex (female/male)	39/35	8/5	Female
Intractable diarrhea	76/77	14/14	+
Facial dysmorphism	66/67	10/10	+
Hair abnormalities	71/73	11/13	+
Trichorrhexis nodosa	46/59	5/13	+
Immunodeficiency	48/67	5/12	−
IUGR/SGA	48/63	9/9	+
Liver disease	41/61	8/10	−
Skin abnormalities	29/48	6/6	−
Hypo/hyperpigmentation	17/29	4/6	−
Cardiac abnormalities	15/43	4/4	−
Outcome (alive/dead)	56/24	13/1	Alive

IUGR, intrauterine growth retardation; SGA, small for gestational age.

The patient was diagnosed with premature ovarian failure at the age of 28 years, which has never been reported in patients with THES before. There is still little information concerning the etiologies of such pathologies (Beck-Peccoz and Persani, 2006). Genetic, autoimmune, infectious, metabolic, and iatrogenic factors all appear to potentially play a role. Our proband did not receive abdominal surgery, radiotherapy, or chemotherapy. It was recently reported that exposure to common reproductively toxic environmental chemicals contributes to early menopause and even premature ovarian failure (Ge et al., 2019). Similarly, the unavoidable exposure to these chemicals may have induced premature ovarian failure in our proband. Another postulation is that, as *SKIV2L* is a specific negative regulator of RNA-activated RIG-I-like (RLR) response, her hypergonadotropic hypogonadism may be because patients with *SKIV2L* deficiency have a strong type I interferon (IFN) signature in the blood, indicating a chronic and inappropriate antiviral response and may lead to immune system activation and cellular damage. Mutations in *SKIV2L* may lead to structural loss-of-function to SKI2W (Figure 2), which may lead to the destruction of ovarian cells due to chronic autoimmune response (Eckard et al., 2014). The study only tested two THES patients with biallelic *SKIV2L* mutations; more samples may be required to further evaluate the rationality of the mechanism mentioned above.

After a thorough literature search, we observed that no patient diagnosed with THES presented with osteoporosis. Considering that our proband was diagnosed with premature ovarian failure, we believe that this may be due to long-term adverse effects of estrogen deficiency (Raisz, 2005; European Society for Human et al., 2016; Almeida et al., 2017). Recent studies have suggested that SKIV2L deficiency activates mTORC1, which regulates osteoblast differentiation and activity (Liu et al., 2018; Hiraiwa et al., 2019) and induces T cell hyperactivation (Yang et al., 2022). However, since the results of these studies are inconsistent, further research is required to elucidate the exact mechanism(s) underlying the role of mTORC1 in osteoporosis and THES.

In conclusion, this case report adds to the available information regarding the characteristics of THES. We show that premature ovarian failure may occur in patients with THES and reveal a new example of the phenotypic spectrum of *SKIV2L* mutations. Molecular testing can be considered for THES diagnosis in cases of intractable diarrhea, growth restriction, and amenorrhea.

## Data Availability

The data analyzed in this study is subject to the following licenses/restrictions: The raw datasets generated and/or analyzed during the current study are not publicly available in order to protect the participant’s confidentiality. The data and materials are available from the corresponding author upon reasonable request. Requests to access these datasets should be directed to XS, brento@126.com.

## Abbreviations

THES, Trichohepatoenteric syndrome; TTC37, Tetratricopeptide repeat domain 37; *SKIV2L*, Superkiller viralicidic activity 2-like gene.

## References

[B1] Almeida M. Laurent M. R. Dubois V. Claessens F. O'Brien C. A. Bouillon R. (2017). Estrogens and Androgens in Skeletal Physiology and Pathophysiology. Physiol. Rev. 97, 135–187. 10.1152/physrev.00033.2015 27807202 PMC5539371

[B2] An W. Zhang J. Chang L. Zhang Y. Wan Y. Ren Y. (2015). Mutation Analysis of Chinese Sporadic Congenital Sideroblastic Anemia by Targeted Capture Sequencing. J. Hematol. Oncol. 8, 55. 10.1186/s13045-015-0154-0 25985931 PMC4490691

[B3] Auwera G. A. Carneiro M. O. Hartl C. Poplin R. Del Angel G. Levy‐Moonshine A. (2013). From FastQ Data to High‐Confidence Variant Calls: The Genome Analysis Toolkit Best Practices Pipeline. Curr. Protoc. Bioinformatics 43, 1–33. 10.1002/0471250953.bi1110s43 25431634 PMC4243306

[B4] Beck-Peccoz P. Persani L. (2006). Premature Ovarian Failure. Orphanet J. Rare Dis. 1, 9. 10.1186/1750-1172-1-9 16722528 PMC1502130

[B5] Bertoni M. Kiefer F. Biasini M. Bordoli L. Schwede T. (2017). Modeling Protein Quaternary Structure of Homo- and Hetero-Oligomers beyond Binary Interactions by Homology. Sci. Rep. 7, 10480. 10.1038/s41598-017-09654-8 28874689 PMC5585393

[B6] Bienert S. Waterhouse A. de Beer T. A. P. Tauriello G. Studer G. Bordoli L. (2017). The SWISS-MODEL Repository-New Features and Functionality. Nucleic Acids Res. 45, D313–D319. 10.1093/nar/gkw1132 27899672 PMC5210589

[B7] Bourgeois P. Esteve C. Chaix C. Béroud C. Lévy N. Fabre A. (2018). Tricho-Hepato-Enteric Syndrome Mutation Update: Mutations Spectrum of TTC37 and SKIV2L , Clinical Analysis and Future Prospects. Hum. Mutat. 39, 774–789. 10.1002/humu.23418 29527791

[B8] Chen J. J. Shi L. P. (2017). A Case of Tricho-Hepato-Enteric Syndrome. Zhonghua Er Ke Za Zhi 55, 308–309. 10.3760/cma.j.issn.0578-1310.2017.04.015 28441830

[B9] Chong J. H. Jamuar S. S. Ong C. Thoon K. C. Tan E. S. Lai A. (2015). Tricho-hepato-enteric Syndrome (THE-S): Two Cases and Review of the Literature. Eur. J. Pediatr. 174, 1405–1411. 10.1007/s00431-015-2563-z 25976726

[B10] Eckard S. C. Rice G. I. Fabre A. Badens C. Gray E. E. Hartley J. L. (2014). The *SKIV2L* RNA Exosome Limits Activation of the RIG-I-like Receptors. Nat. Immunol. 15, 839–845. 10.1038/ni.2948 25064072 PMC4139417

[B11] European Society for Human R. Embryology Guideline Group on P. O. I. Webber L. Davies M. Anderson R. Bartlett J. (2016). ESHRE Guideline: Management of Women with Premature Ovarian Insufficiency. Hum. Reprod. 31, 926–937. 10.1093/humrep/dew027 27008889

[B12] Fabre A. Badens C. (2014). Human Mendelian Diseases Related to Abnormalities of the RNA Exosome or its Cofactors. Irdr 3, 8–11. 10.5582/irdr.3.8 25343120 PMC4204543

[B13] Fabre A. Bourgeois P. Chaix C. Bertaux K. Goulet O. Badens C. (2018). "Trichohepatoenteric Syndrome," in GeneReviews(R), Editos. Adam M. P. Ardinger H. H. Pagon R. A. Wallace S. E. Bean L. J. H. Mirzaa G. (Seattle WA), 1–10. 29334452

[B14] Fabre A. Bourgeois P. Coste M.-E. Roman C. Barlogis V. Badens C. (2017). Management of Syndromic Diarrhea/tricho-Hepato-Enteric Syndrome: A Review of the Literature. Irdr 6, 152–157. 10.5582/irdr.2017.01040 28944135 PMC5608923

[B15] Fabre A. Charroux B. Martinez-Vinson C. Roquelaure B. Odul E. Sayar E. (2012). *SKIV2L* Mutations Cause Syndromic Diarrhea, or Trichohepatoenteric Syndrome. Am. J. Hum. Genet. 90, 689–692. 10.1016/j.ajhg.2012.02.009 22444670 PMC3322239

[B16] Ge W. Li L. Dyce P. W. De Felici M. Shen W. (2019). Establishment and Depletion of the Ovarian reserve: Physiology and Impact of Environmental Chemicals. Cell. Mol. Life Sci. 76, 1729–1746. 10.1007/s00018-019-03028-1 30810760 PMC11105173

[B38] Girault D. Goulet O. Le Deist F. Brousse N. Colomb V. Cesarini J. P. (1994). Intractable Infant Diarrhea Associated With Phenotypic Abnormalities and Immunodeficiency. J. Pediatr. 125, 36–42. 10.1016/s0022-3476(94)70118-0 8021782

[B17] Guex N. Peitsch M. C. Schwede T. (2009). Automated Comparative Protein Structure Modeling with SWISS-MODEL and Swiss-PdbViewer: a Historical Perspective. Electrophoresis 30 (Suppl. 1), S162–S173. 10.1002/elps.200900140 19517507

[B18] Hartley J. L. Zachos N. C. Dawood B. Donowitz M. Forman J. Pollitt R. J. (2010). Mutations in *TTC37* Cause Trichohepatoenteric Syndrome (Phenotypic Diarrhea of Infancy). Gastroenterology 138, 2388–2398. 10.1053/j.gastro.2010.02.010 20176027 PMC3166659

[B19] Hiraiwa M. Ozaki K. Yamada T. Iezaki T. Park G. Fukasawa K. (2019). mTORC1 Activation in Osteoclasts Prevents Bone Loss in a Mouse Model of Osteoporosis. Front. Pharmacol. 10, 684. 10.3389/fphar.2019.00684 31263418 PMC6585391

[B20] Karaca Edeer N. Aykut A. Pariltay E. Aksu G. Cogulu O. Kutukculer N. (2019). A Novel *TTC37* Mutation Causing Clinical Symptoms of Trichohepatoenteric Syndrome Such as Pyoderma Gangrenosum and Immunodeficiency without Severe Diarrhea. J. Investig. Allergol. Clin. Immunol. 29, 396–398. 10.18176/jiaci.0418 31132033

[B21] Kechin A. Boyarskikh U. Kel A. Filipenko M. (2017). cutPrimers: A New Tool for Accurate Cutting of Primers from Reads of Targeted Next Generation Sequencing. J. Comput. Biol. 24, 1138–1143. 10.1089/cmb.2017.0096 28715235

[B22] Kögel A. Keidel A. Bonneau F. Schäfer I. B. Conti E. (2022). The Human SKI Complex Regulates Channeling of Ribosome-Bound RNA to the Exosome via an Intrinsic Gatekeeping Mechanism. Mol. Cel. 82, 756–769. e758. 10.1016/j.molcel.2022.01.009 PMC8860381 35120588

[B23] Lee W.-I. Huang J.-L. Chen C.-C. Lin J.-L. Wu R.-C. Jaing T.-H. (2016a). Identifying Mutations of the Tetratricopeptide Repeat Domain 37 (TTC37) Gene in Infants with Intractable Diarrhea and a Comparison of Asian and Non-asian Phenotype and Genotype. Medicine (Baltimore) 95, e2918. 10.1097/MD.0000000000002918 26945392 PMC4782876

[B24] Lee W. S. Teo K. M. Ng R. T. Chong S. Y. Kee B. P. Chua K. H. (2016b). Novel Mutations in *SKIV2L* and *TTC37* Genes in Malaysian Children with Trichohepatoenteric Syndrome. Gene 586, 1–6. 10.1016/j.gene.2016.03.049 27050310

[B25] Li H. Durbin R. (2009). Fast and Accurate Short Read Alignment with Burrows-Wheeler Transform. Bioinformatics 25, 1754–1760. 10.1093/bioinformatics/btp324 19451168 PMC2705234

[B26] Liu Q. Liu C. Yang Y. Yang H. Chen J. (2018). Osteocyte‐intrinsic mTORC1 Signaling Restrains Trabecular Bone Accrual in Mice. J. Cel. Biochem. 119, 8743–8749. 10.1002/jcb.27470 30160781

[B27] Poulton C. Pathak G. Mina K. Lassman T. Azmanov D. N. McCormack E. (2019). Tricho-hepatic-enteric Syndrome (THES) without Intractable Diarrhoea. Gene 699, 110–114. 10.1016/j.gene.2019.02.059 30844479 PMC7872052

[B28] Raisz L. G. (2005). Pathogenesis of Osteoporosis: Concepts, Conflicts, and Prospects. J. Clin. Invest. 115, 3318–3325. 10.1172/JCI27071 16322775 PMC1297264

[B29] Richards S. Aziz N. Bale S. Bick D. Das S. Gastier-Foster J. (2015). Standards and Guidelines for the Interpretation of Sequence Variants: a Joint Consensus Recommendation of the American College of Medical Genetics and Genomics and the Association for Molecular Pathology. Genet. Med. 17, 405–424. 10.1038/gim.2015.30 25741868 PMC4544753

[B30] Rider N. L. Boisson B. Jyonouchi S. Hanson E. P. Rosenzweig S. D. Cassanova J.-L. (2015). Novel *TTC37* Mutations in a Patient with Immunodeficiency without Diarrhea: Extending the Phenotype of Trichohepatoenteric Syndrome. Front. Pediatr. 3, 2. 10.3389/fped.2015.00002 25688341 PMC4311608

[B31] Stankler L. Lloyd D. Pollitt R. J. Gray E. S. Thom H. Russell G. (1982). Unexplained Diarrhoea and Failure to Thrive in 2 Siblings with Unusual Facies and Abnormal Scalp Hair Shafts: a New Syndrome. Arch. Dis. Child. 57, 212–216. 10.1136/adc.57.3.212 7073301 PMC1627586

[B32] Studer G. Rempfer C. Waterhouse A. M. Gumienny R. Haas J. Schwede T. (2020). QMEANDisCo-Distance Constraints Applied on Model Quality Estimation. Bioinformatics 36, 1765–1771. 10.1093/bioinformatics/btz828 31697312 PMC7075525

[B33] Wang K. Li M. Hakonarson H. (2010). ANNOVAR: Functional Annotation of Genetic Variants from High-Throughput Sequencing Data. Nucleic Acids Res. 38, e164. 10.1093/nar/gkq603 20601685 PMC2938201

[B34] Waterhouse A. Bertoni M. Bienert S. Studer G. Tauriello G. Gumienny R. (2018). SWISS-MODEL: Homology Modelling of Protein Structures and Complexes. Nucleic Acids Res. 46, W296–W303. 10.1093/nar/gky427 29788355 PMC6030848

[B35] Yang K. Han J. Asada M. Gill J. G. Park J. Y. Sathe M. N. (2022). Cytoplasmic RNA Quality Control Failure Engages mTORC1-Mediated Autoinflammatory Disease. J. Clin. Invest. 132. 10.1172/JCI146176 PMC8759780 35040435

[B36] Zhang Q. Qian X. Zhou J. Han L. Zhou S. Wang Z. (2021). Case Report: Novel Compound-Heterozygous Variants of *SKIV2L* Gene that Cause Trichohepatoenteric Syndrome 2. Front. Genet. 12, 756451. 10.3389/fgene.2021.756451 34691159 PMC8527088

[B37] Zheng B. Pan J. Jin Y. Wang C. Liu Z. (2016). Targeted Next-Generation Sequencing Identification of a Novel Missense Mutation of the *SKIV2L* Gene in a Patient with Trichohepatoenteric Syndrome. Mol. Med. Rep. 14, 2107–2110. 10.3892/mmr.2016.5503 27431780

